# Autoimmune comorbidities in patients with metastatic melanoma: a retrospective analysis of us claims data

**DOI:** 10.1186/s12885-018-4051-0

**Published:** 2018-02-06

**Authors:** Qiufei Ma, Mark Shilkrut, Zhongyun Zhao, Minming Li, Nicolas Batty, Beth Barber

**Affiliations:** 1 Department of Global Health Economics, Amgen Inc., One Amgen Center Drive, Thousand Oaks, CA 91320-1799 USA; 2 Clinical Research Oncology/Hematology, Amgen Inc., Thousand Oaks, CA USA; 3 Department of Biostatistics, University of Massachusetts at Amherst, Amherst, MA USA

**Keywords:** Autoimmune, Comorbidities, Metastatic melanoma, Retrospective analysis, Prevalence rate

## Abstract

**Background:**

Immunotherapies have advanced the treatment of metastatic melanoma; however, they are associated with immune-related toxicities. Patients with pre-existing autoimmune comorbidities are commonly excluded from clinical trials investigating immunotherapies in metastatic melanoma. Since information on pre-existing autoimmune comorbidities in “real-world” patients with newly diagnosed metastatic melanoma is limited, we sought to estimate the prevalence of autoimmune comorbidities and its change over time.

**Methods:**

Data were obtained from a large US claims database, MarketScan®, from 2004 to 2014. Records of patients with newly diagnosed metastatic or non-metastatic melanoma and of general population were analyzed. Autoimmune comorbidities were defined as presence of autoimmune disorders, which were obtained from the list of diseases at the American Autoimmune-Related Diseases Association web portal (www.aarda.org). The prevalence of pre-existing autoimmune comorbidities and its change over the 11-year period were calculated. Logistic regression analyses were performed to evaluate the relationship between clinical and demographic factors and pre-existing autoimmune comorbidities in patients with metastatic melanoma.

**Results:**

This study assessed the prevalence and change of prevalence over a period of 11 years of 147 autoimmune comorbidities. Among 12,028 patients with newly diagnosed metastatic melanoma, the prevalence rate of pre-existing autoimmune comorbidities increased from 17.1% in 2004 to 28.3% in 2014 (*P* < 0.001). The prevalence rates of autoimmune comorbidities increased from 11.7% in 2004 to 19.8% in 2014 in patients with non-metastatic melanoma and 7.9% in 2004 to 9.2% in 2014 in the general population. In addition, patients with bone or gastrointestinal melanoma metastases, those with more comorbid diseases, or female patients, were found to have a higher risk of autoimmune comorbidities.

**Conclusions:**

The prevalence of pre-existing autoimmune comorbidities in patients with newly diagnosed metastatic melanoma was high, and increased over 11 years. In comparison, a lower prevalence of autoimmune comorbidities was seen in patients with newly diagnosed non-metastatic melanoma and in the general population. Increases in prevalence for these population groups were also observed over 11 years. Impact of autoimmune comorbidities on treatment decisions in patients with metastatic melanoma should be explored.

## Background

More than 73,000 new cases of melanoma were expected to be diagnosed in 2015. This represents less than 2% of all new skin cancer cases; however, such cases are responsible for 90% of skin cancer-related deaths [1, 2].

Treatment guidelines for melanoma reflect both the biological characteristics of the tumor and the stage at which it is detected. If discovered early, excision represents the standard of care for early-stage melanoma [3]. Surgical removal is also recommended for loco-regional recurrences of melanoma. For distant metastases, surgery may also be considered if the disease is site- and volume-limited and is technically resectable; however, for unresectable metastatic disease current guidelines recommend: systemic therapy, clinical trial, intralesional injection, or best supportive care (recommendations differ for different stages, biological characteristics of the tumor, performance status of the patient etc.) [3].

US cancer statistics show 5-year relative survival rate for localized melanoma (84% of cases) of 98%; however, survival declines to 63% and 16% for regional and distant-stage disease, respectively [1]. Real-world data from patients with metastatic melanoma have shown great variation in survival time according to disease stage of the melanoma [4]. In a study of patients with metastatic melanoma from the Surveillance, Epidemiology, and End Results database [5], the median overall survival time (time between the date of randomization and the date of death) was longer in patients diagnosed with unresectable non-visceral (stage IIIB/C or IVM1a) metastatic melanoma (22.3–24.3 months) than in patients diagnosed with visceral unresectable stage IVM1b or IVM1c metastatic melanoma (5.1–11.2 months) [5].

Prior to 2011, some improvement in response rates were observed with systemic biochemotherapy (cytokine-based immunotherapy in combination with cytotoxic chemotherapy) [6–8]. However, treatment options for metastatic melanoma prior to 2011 offered little hope for patients with metastatic melanoma as none of the available options had demonstrated an effect on overall survival. Advances in the development of cancer immunotherapies included insights into the mechanisms used by cancer cells to subvert the antitumor immune response, including the production of inhibitory cytokines, recruitment of immunosuppressive immune cells and the upregulation of co-inhibitory receptors known as immune checkpoints [9]. As a result, some success has been achieved using immunotherapies targeting cancer-associated immunosuppression; notably, the use of immune checkpoint inhibitors [9]. These findings, in addition to the discovery of melanoma driver mutation subsets and the important role that the mitogen-activated protein kinase pathway plays in a significant part of melanomas [10], have led to the approval of nine new treatments or combinations thereof since 2011 [3, 9, 11].

Among the treatments approved since 2011, B-Raf proto-oncogene, serine/threonine kinase and mitogen-activated protein kinase 1 inhibitors target abnormally activated protein kinases [11], whereas immune checkpoint inhibitors act by enhancing T-lymphocyte function. Regulatory approved immune checkpoint inhibitors include ipilimumab, pembrolizumab, and nivolumab, while molecularly targeted drugs approved at this time include vemurafenib, dabrafenib and trametinib and cobimetinib, used as single agents (first three) or in combination.

The use of immune checkpoint inhibition has led to significant improvements in some clinical outcomes for patients with metastatic melanoma, giving rise to approvals of ipilimumab, nivolumab, and pembrolizumab as single agents in this setting. Current clinical trials focus on the combination of immune checkpoint inhibitors with each other, or with targeted therapies. However; to date, the optimal sequence and combination of targeted drugs and immunotherapies are still unknown [11].

There are limitations to the use of immunotherapy, with immune-related adverse events (AEs) associated with these treatments [12, 13]. Ipilimumab therapy has been associated with distinctive immune-related AEs, including colitis, dermatitis, hepatitis, endocrinopathies and neuropathies, all of which may be attributed to an inflammatory effect of non-specific lymphocyte activation [11]. Of patients treated with ipilimumab, 10%–15% have been shown to experience immune-related AEs of grade 3 or higher [11]. In a phase 3 study assessing ipilimumab as adjuvant therapy for patients with completely resected stage III melanoma [14], five patients died due to ipilimumab-related AEs. Results from a phase 2 study of pembrolizumab in advanced melanoma reported a less common incidence of immune-mediated AEs and of lesser severity [15]. These immune AEs occur despite clinical trial protocols that exclude patients with pre-existing autoimmune comorbidities.

The approval label for immune checkpoint inhibitors reflects the potential occurrence of immune-related AEs with these agents; the US Food and Drug Administration Prescribing Information and European Medicines Agency Summary of Product Characteristics for ipilimumab, nivolumab and pembrolizumab carry warnings that these agents are associated with immune-related AEs [16–21]. Patient information brochures inform recipients of these risks; in addition, risk management plans have been developed for these agents to ensure that they are used as safely as possible [22–24].

Patients with active, known or suspected autoimmune disorders were thought to be at greater risk of developing immune-mediated AEs and would therefore not be good candidates for inclusion in clinical studies of immune checkpoint inhibitors; they were excluded from the pivotal randomized controlled trials of these treatments [25–27]. Hence, the clinical data do not provide an overall indication of the prevalence of autoimmune disorders within the population of patients with metastatic melanoma nor the extent of the patient population for whom immune-related AEs represent a barrier to the use of immune checkpoint inhibition.

Additionally, limited information is available as to the prevalence of patients with metastatic melanoma who also have pre-existing autoimmune disorders. This study therefore aims to assess the prevalence of autoimmune disorders and time trend over a period of 11 years from 2004 to 2014 primarily in patients with metastatic melanoma.

## Methods

### Study design and data source

This is a US population-based retrospective study using claims data from the Truven MarketScan® database. This database contains information on approximately 30 million people with employer-sponsored primary or Medicare supplemental insurance. The database includes dates of enrollment in the insurance program, inpatient and outpatient visits, and prescription drugs dispensed. Demographic data include sex, age, and region. All claims were coded with International Classification of Diseases, Ninth Edition (ICD-9), codes [28].

### Study measures

A list of autoimmune disorders assessed in this study were obtained from the site of the American Autoimmune-Related Diseases Association (www.aarda.org). All 147 autoimmune conditions posted at their site were included [29]. For each condition, corresponding ICD-9-CM codes were used to identify the condition in the Truven MarketScan® database.

The following covariates for the prevalence of autoimmune comorbidities were evaluated for the patients with metastatic melanoma: demographic factors (age, gender and geographical region), metastatic site, Charlson Comorbidity Score (a weighted score of 16 pre-existing diseases, excluding cancers) and Category (whether the score is 0, 1, or > = 2), and health insurance plan, including being a Medicare beneficiary.

### Study populations

Records between July 1, 2003 and June 30, 2014 were obtained from the Truven MarketScan® Commercial and Medicare database. Three population groups were defined for analysis.

The first group were patients with metastatic melanoma with at least one inpatient or outpatient melanoma diagnosis (ICD-9-CM: 172.xx, V10.82) and at least one inpatient or outpatient metastatic diagnosis (ICD-9-CM: 196.xx-198.xx). The first metastasis diagnosis date should be less than 30 days before, or any time after, the melanoma diagnosis. The index date was the first date of metastasis diagnosis. An autoimmune comorbidity was indicated as “present” for a given patient if any of the autoimmune conditions codes were detected during the 12 months prior to or on the index date.

The second patient group comprised those with non-metastatic melanoma. The index date was the first date of melanoma diagnosis and those patients who subsequently developed metastases were excluded. An autoimmune comorbidity was indicated as “present” if any of the autoimmune conditions codes were detected during the 12 months prior to or on the index date.

Of note, patients with metastatic melanoma or non-metastatic melanoma were required to be continuously enrolled in the health plan for at least 1 year prior to the index date. Please also note that each patient could only appear once in the metastatic melanoma or non-metastatic melanoma cohort.

Lastly, a general population group included all people in the database who were beneficiaries between January 1, 2004, and June 30, 2014. For each year, autoimmune comorbidity was defined as one or more of the 147 autoimmune conditions occurring within that year.

### Statistical analysis

Descriptive analyses were conducted to estimate the prevalence of autoimmune comorbidities within each of the three populations described. The trend in prevalence over a period of 11 years from 2004 to 2014 was assessed.

Clinical and demographic characteristics were compared between patients with metastatic melanoma who had autoimmune comorbidities and those who did not have. T-tests were performed for continuous variables and a chi-square test for categorical variables. Logistic regression analysis was conducted to determine if a statistically significant change had occurred for the prevalence of autoimmune comorbidities among patients with metastatic melanoma. Multivariate logistic regressions were used to examine the impact of covariates on autoimmune comorbidities in patients with metastatic melanoma. Odds ratios (ORs) with 95% confidence intervals (CIs) were presented.

All data were extracted and analyzed using programs organized within SAS® Enterprise Guide version 4.2 (SAS Institute Inc., Cary, NC, USA) and conducted under UNIX using SAS version 9.2. All statistical tests were 2-sided.

## Results

### Common Autoimmune comorbidities

Among 147 autoimmune conditions that were assessed in patients with metastatic melanoma, the ten most prevalent are shown in Table 1.

**Table 1:** The ten most prevalent autoimmune conditions in individuals with metastatic melanoma

No.	Autoimmune conditions	ICD-9 codes	% in metastatic melanoma (*N* = 12,028)	% in non-metastatic melanoma (*N* = 127,184)	% in general population (*N* = 342,492,007)
1	Myositis	729.1	3.6	3.1	2.2
2	Peripheral neuropathy	356.0, 356.8, 356.9	2.6	1.5	0.5
3	Type 1 diabetes mellitus	250.×1, 250.×3.	2.4	1.6	1.1
4	Rheumatoid arthritis	714, 714.0, 714.1, 714.2, 714.3, 714.31, 714.32, 714.33, 714.4, 714.8, 714.81, 714.89, 714.9	1.7	1.4	0.8
5	Psoriasis	696, 696.0, 696.1, 696.2, 696.3, 696.4, 696.5, 696.8	1.1	1.5	0.8
6	Autoimmune pancreatitis	577.0, 577.1	1.1	0.3	0.2
7	Autoimmune aplastic anemia	284, 284.89, 284.9	1.0	0.1	< 0.1
8	Relapsing polychondritis	733.99	0.9	0.3	0.2
9	Hashimoto’s encephalitis	348.30	0.9	0.2	0.2
10	Ulcerative colitis	556, 556.0, 556.1, 556.2, 556.3, 556.4, 556.5, 556.6, 556.8, 556.9	0.7	0.5	0.3

### Characteristics in patients with metastatic melanoma

A total of 12,028 patients with newly diagnosed metastatic melanoma were identified in the database: 2980 had autoimmune comorbidities and 9048 did not have autoimmune comorbidities. Demographic and clinical characteristics of these two groups of patients are shown in Table 2.

**Table 2:** Demographic and clinical characteristics of individuals with metastatic melanoma

	Total (*N* = 12,028)	Without autoimmune conditions (*n* = 9048)	With autoimmune conditions (*n* = 2980)	*P-*value
Age, mean (SD)^a^	64.2 (14.2)	63.9 (14.4)	65.2 (13.6)	< 0.0001
Age group, n (%)^a^				< 0.0001
18–34	289 (2.4)	235 (2.6)	54 (1.8)	
35–54	2614 (21.7)	2049 (22.7)	565 (19.0)	
55–64	3553 (29.5)	2651 (29.3)	902 (30.3)	
65–74	2304 (19.2)	1681 (18.6)	623 (20.9)	
≥ 75	3268 (27.2)	2432 (26.9)	836 (28.1)	
Gender, n (%)^a^				< 0.0001
Male	7574 (63.0)	5824 (64.4)	1750 (58.7)	
Female	4454 (37.0)	3224 (35.6)	1230 (41.3)	
Health insurance plan, n (%)^a^				0.042
Comprehensive	2799 (23.3)	2109 (23.3)	690 (23.2)	
HMO	1429 (11.9)	1120 (12.4)	309 (10.4)	
POS	578 (4.8)	438 (4.8)	140 (4.7)	
PPO	6058 (50.4)	4520 (50.0)	1538 (51.6)	
Others	1164 (9.7)	861 (9.5)	303 (10.2)	
Medicare beneficiaries, n (%)^a^				0.007
Yes	5517 (45.9)	4086 (45.2)	1431 (48.0)	
No	6511 (54.1)	4962 (54.8)	1549 (52.0)	
Regions, n (%)				0.109
North East	1760 (14.6)	1296 (14.3)	464 (15.6)	
North Central	3065 (25.5)	2320 (25.6)	745 (25.0)	
South	4165 (34.6)	3180 (35.2)	985 (33.1)	
West	2549 (21.2)	1888 (20.9)	661 (22.2)	
Unknown/Other	489 (4.1)	364 (4.0)	125 (4.2)	
Metastasis site, n (%)^a^				< 0.0001
Brain	2082 (17.3)	1551 (17.1)	531 (17.8)	
Bone	1541 (12.8)	1044 (11.5)	497 (16.7)	
Liver	1109 (9.2)	814 (9.0)	295 (9.9)	
Gastro-intestinal system	386 (3.2)	269 (3.0)	117 (3.9)	
Lungs	1372 (11.4)	1042(11.5)	330 (11.1)	
Charlson comorbidity score, mean (SD)^a^				< 0.0001
	0.6 (1.0)	0.5 (0.9)	0.9 (1.3)	
Charlson Category, N (%)^a^				< 0.0001
0	8038 (66.8)	6477 (71.6)	1561 (52.4)	
1	2430 (20.2)	1687 (18.7)	743 (24.9)	
≥ 2	1560 (13.0)	884 (9.8)	676 (22.7)	

*HMO* health maintenance organization, *POS* point of service plan, *PPO* preferred provider organization, *SD* standard deviation

^a^indicates statistically significant at 5%

The distribution of age, gender, health plan type, Medicare beneficiaries, metastatic site, and Charlson Comorbidity Index score or category were different between the individuals with autoimmune comorbidities versus those without autoimmune comorbidities. Individuals with autoimmune comorbidities had a higher proportion of females, a higher proportion of individuals older than 65, and a higher mean Charlson comorbidity score.

### Prevalence trends of Autoimmune comorbidities between 2004 and 2014

Among 12,028 patients with newly diagnosed metastatic melanoma, the prevalence rate of autoimmune comorbidities increased 1.7-fold: from 17.1% in 2004 to 28.3% in 2014, and the time trend was statistically significant (*P* < 0.001) based on a trend analysis using a GENMOD regression using the whole data.

**Fig. 1:**
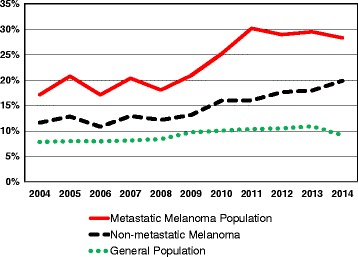
One-year prevalence of autoimmune comorbidities in individuals with metastatic melanoma, non-metastatic melanoma, and in the general population, from 2004 to 2014

The prevalence rates of autoimmune comorbidities were lower in patients with non-metastatic melanoma (11.7% in 2004 to 19.8% in 2014) and in the general population (7.9% in 2004 to 9.2% in 2014), but the rates increased 1.7- and 1.2-fold over time, respectively (Fig. 1; Table 3).

**Table 3:** Total yearly patient numbers derived from the database

Year	Metastatic melanoma population	Non-metastatic melanoma population	General population
2004	496	5526	19,126,506
2005	756	7278	21,519,205
2006	590	5906	23,368,796
2007	889	8210	23,494,037
2008	1047	10,738	28,532,791
2009	1325	16,101	33,672,641
2010	883	12,333	37,437,295
2011	1515	18,493	44,028,795
2012	1828	20,660	44,745,958
2013	1522	17,784	37,307,087
2014	1177	4155	29,258,896
Total	12,028	127,184	342,492,007

### Factors influencing autoimmune comorbidities for patients with metastatic melanoma

Multivariate logistic regression analysis of covariates/factors for prevalence of autoimmune comorbidities in patients with metastatic melanoma is shown in Table 4. Patients were more likely to have autoimmune comorbidities if they were females (vs. males; OR = 1.42; 95% CI: 1.30–1.55), had bone metastasis (vs. skin metastases, OR = 1.77; 95% CI: 1.37–2.29), had gastrointestinal metastases (vs. skin metastases, OR = 1.64; 95% CI: 1.19–2.27), or had a higher Charlson comorbidity score (vs. score being zero, OR = 3.37; 95% CI: 2.99–3.80).

**Table 4:** Multivariate logistic regression of factors for autoimmune comorbidities in individuals with metastatic melanoma

	Odds ratio	95% confidence interval		*P*-value
		Lower	Upper	
Age group (Reference: 55–64)				
18–34	0.83	0.61	1.14	0.25
35–54	0.90	0.79	1.02	0.09
65–74	1.09	0.87	1.37	0.46
≥ 75	0.92	0.72	1.17	0.48
Female^a^ (Reference: Male)	1.42	1.30	1.55	< 0.001
Health Insurance Plan (Reference: Others)				
Comprehensive	0.84	0.70	1.00	0.06
HMO^a^	0.75	0.62	0.90	0.01
POS	0.92	0.72	1.17	0.51
PPO	0.98	0.85	1.14	0.83
Medicare beneficiaries (Reference: No Medicare)	0.94	0.75	1.19	0.62
Regions (Reference: West)				
North East	0.96	0.83	1.11	0.57
North Central^a^	0.88	0.78	0.99	0.04
South^a^	0.86	0.76	0.96	0.01
Metastasis site at index (Reference: Skin)				
Brain	1.22	0.95	1.57	0.12
Bone^a^	1.77	1.37	2.29	< 0.001
Liver	1.30	1.00	1.71	0.05
Gastrointestinal^a^	1.64	1.19	2.27	0.01
Lung	1.19	0.91	1.55	0.20
Charlson Category (Reference: 0)				
1^a^	1.88	1.69	2.09	< 0.001
≥ 2^a^	3.37	2.99	3.80	< 0.001

*HMO* health maintenance organization, *POS* point of service plan, *PPO* preferred provider organization

^a^Indicates statistically significant at 5%

### Sensitivity analyses

Sensitivity analyses were carried out in patients with metastatic renal-cell carcinoma (RCC) and metastatic breast cancer, respectively. Prevalence rates of autoimmune comorbidities increased from 18.5% to 28.3% for RCC and from 17.7% to 24.2% for metastatic breast cancer between 2004 and 2014. The increase in prevalence of autoimmune disease in patients with metastatic melanoma (from 17.1% to 28.3%; 2004–2014) is similar to that found for patients with RCC. On the other hand, metastatic breast cancer has slightly lower prevalence of autoimmune diseases and extent at which their occurrence increased over the study duration in patients with this tumor type compared with patients with melanoma.

## Discussion

More recently approved treatments for metastatic melanoma, such as immune checkpoint inhibitors, have been shown to be associated with immune-related AEs. Therefore, pivotal clinical studies undertaken with these agents excluded patients with immune-related disorders. This study estimated the prevalence of immune-related comorbidities in patients with metastatic melanoma using existing claims data gathered between January 1, 2004 and June 30, 2014.

The study analyzed three patient populations: patients with newly metastatic melanoma, patients with non-metastatic melanoma, and the general population. Over the study period the prevalence of autoimmune comorbidities in all three patient groups increased over time from 2004 to 2014. The prevalence of autoimmune comorbidities in patients with metastatic melanoma is higher than that in patients with non-metastatic melanoma, and the prevalence in both these patient groups is higher than that for the general population. The prevalence of autoimmune disease in the general population in this study of 7.9–9.2% was broadly consistent with existing data on the prevalence of autoimmune disorders in the general population: (7.6 to 9.4%, depending on the size of the correction factor used) [30–32].

Sensitivity analyses were conducted to explore the prevalence of autoimmune comorbidities in RCC and in metastatic breast cancer. The findings in RCC, another cancer that has been considered responsive to immune therapy and associated with autoimmune disorders [33], were consistent with that in metastatic melanoma; while the prevalence and increase over time in metastatic breast cancer were slightly lower than that in metastatic melanoma.

Reasons for the increase in prevalence of autoimmune comorbidities observed in patients with metastatic melanoma compared with patients with melanoma or the general population could include paraneoplastic effects of melanoma on the immune system. In addition, this increase could be due to increased awareness and better diagnosis of immune disorders over the time frame of the study. However, one cannot exclude the possibility that disturbances in shared genetic or immune pathways could lead to increased susceptibility to develop neoplasms in patients afflicted by autoimmune conditions. Additionally, potential use of immunosuppressive drug therapy before or after metastatic melanoma might also result in the increase in auto-immune disease. However, the use of immunosuppressive drug therapy before metastatic melanoma diagnosis was very limited as most patients with in situ or early melanoma will be cured by primary excision alone; and the newer immunotherapies for metastatic melanoma were approved for use only after 2011 (one drug) and 2014 (two drugs).

Knowledge of the likelihood of developing autoimmune disorders or immune-related AEs has implications when choosing treatments for patients and allows greater individualization of treatment. As immunomodulatory treatments such as immune checkpoint inhibitors exert their effects via the immune system, knowledge that patients have comorbid autoimmune disorders could be used to assess their suitability for treatment with such drug classes.

This study also conducted multivariate logistic regression analyses to examine factors for influencing the prevalence of autoimmune comorbidities within the population of patients with metastatic melanoma. This analysis showed that in patients with metastatic melanoma, those who were female, located in South or North Central US regions, had bone or gastrointestinal metastasis, or a high comorbid disease burden (indicated by their Charlson Comorbidity Index), were more likely to have autoimmune comorbidities. These indicated additional factors, which could be taken into account when deciding upon an individual’s course of treatment for metastatic melanoma and their risks of autoimmune comorbidities; and hence whether this would influence the choice of a drug class that acts via the immune system.

Data for this study were taken from an existing database. A limitation of the study is that the data were derived from patients with commercial insurance; therefore, the results may not be representative of all patients, especially those uninsured or covered by Medicaid. ICD-9 codes used to identify metastatic may include metastases due to primary malignant neoplasms other than melanoma. Therefore those metastatic melanoma patients might have other malignant neoplasms. ICD-9 codes for several autoimmune conditions (e.g. Castleman’s disease) explored are not available; and only 12-month prior history was examined for these lifelong autoimmune conditions. Therefore, the true prevalence of autoimmune comorbidities in this study may be underestimated. Additionally, only half year data was available for 2003 and 2014, the prevalence rates of autoimmune diseases for patients with metastatic melanoma or with non-metastatic melanoma might be underestimated for 2004, while the prevalence rate for general population could be underestimated for 2014. Finally, the prevalence estimates are based on diagnoses reported in the claims and hence may also be an underestimate of the true prevalence.

This study has some strengths. This is the first study, to our best knowledge, that examined the prevalence of autoimmune diseases in patients with metastatic melanoma, which is important as newer treatment options for metastatic melanoma, such as checkpoint inhibitors, could induce or worsen autoimmune conditions. The database used for this study contains over 30 million distinct individuals (over 342 million patient-years) and patients enrolled in this database have a similar overall age distribution to the nationally representative population in Medical Expenditure Panel Survey. Finally, sensitivity analyses of prevalence of autoimmune diseases in patients with RCC and metastatic breast cancer provided consistent results as observed in melanoma populations.

## Conclusions

The results of this study showed that the prevalence of autoimmune comorbidities in patients with metastatic melanoma was higher than that in patients with non-metastatic melanoma and in the general population, and had increased substantially over time from 2004 to 2014.

In addition, for metastatic melanoma, those patients who were female, or who were located in South or North Central US regions, with bone or gastrointestinal metastasis, or those patients who had a higher comorbid disease burden were shown to have an increased likelihood of having autoimmune disorders.

These findings of increased prevalence of autoimmune comorbidities among patients with metastatic melanoma are particularly important given that some of the newer agents for treating metastatic melanoma (e.g., checkpoint inhibitors) are associated with a high incidence of severe immune-related AEs. Thus, autoimmune comorbidities in patients with metastatic melanoma should be taken into consideration when treatment decisions are made.

## Abbreviations

AE: Adverse event
CI: Confidence interval
ICD-9: International Classification of Diseases, Ninth Edition
OR: Odds ratio
RCC: Renal-cell carcinoma
